# Discrimination of transgenic soybean seeds by terahertz spectroscopy

**DOI:** 10.1038/srep35799

**Published:** 2016-10-26

**Authors:** Wei Liu, Changhong Liu, Feng Chen, Jianbo Yang, Lei Zheng

**Affiliations:** 1 School of Biotechnology and Food Engineering, Hefei University of Technology, Hefei 230009, China; 2 Intelligent Control and Compute Vision Lab, Hefei University, Hefei 230601, China; 3 Department of Food, Nutrition and Packaging Sciences, Clemson University, Clemson, SC 29634, United States; 4 Rice Research Institute, Anhui Academy of Agricultural Sciences, Hefei 230031, China; 5 School of Medical Engineering, Hefei University of Technology, Hefei 230009, China

## Abstract

Discrimination of genetically modified organisms is increasingly demanded by legislation and consumers worldwide. The feasibility of a non-destructive discrimination of glyphosate-resistant and conventional soybean seeds and their hybrid descendants was examined by terahertz time-domain spectroscopy system combined with chemometrics. Principal component analysis (PCA), least squares-support vector machines (LS-SVM) and PCA-back propagation neural network (PCA-BPNN) models with the first and second derivative and standard normal variate (SNV) transformation pre-treatments were applied to classify soybean seeds based on genotype. Results demonstrated clear differences among glyphosate-resistant, hybrid descendants and conventional non-transformed soybean seeds could easily be visualized with an excellent classification (accuracy was 88.33% in validation set) using the LS-SVM and the spectra with SNV pre-treatment. The results indicated that THz spectroscopy techniques together with chemometrics would be a promising technique to distinguish transgenic soybean seeds from non-transformed seeds with high efficiency and without any major sample preparation.

Soybean (*Glycine max* L.) is one of the most important crops in the world that provides high amounts of oils, proteins, and biologically active components. Genetically modified (GM) soybean has made rapid strides in the past decades and the GM cultivation area is increasing year by year. The ROUNDUP READY (RR) soybean which incorporates a gene from the bacteria *Agrobacterium tumefaciens* conferring resistance to the glyphosate-based ROUNDUP herbicide, now rapidly displaced conventional soybeans because of advantages for crop management and yields. Of the global 90 million hectares of soybean grown in 2010, an impressive 73.3 million hectares were RR soybean^1^. Despite the widely recognized benefits of GM organisms, governmental organizations and/or the general public are still hesitant or opposed to the cultivation and application of GM organisms for a variety of reasons, such as their impact on human and animal health, effects on the environment, and socioeconomic effects. Particularly in the case of crop-to-crop gene flow, as more transgenic cultivars become available, the contamination of conventional cultivars with transgenes and the unintended combination of transgenes through natural hybridization will become increasingly probable.

Gene flow is a natural phenomenon that normally occurs via pollen but can also be caused by natural or human movement during production or commercialization of seeds and vegetative propagules. Soybean is an annual self-pollinating species where pollination occurs either in the bud stage or before flowers completely open. Soybean pollen is too heavy for wind transport but pollination by honey bees, thrips species and predatory Hemiptera has been shown to increase the possibility of out-crossing^2^,^3^. Consequently, out-crossing with pollen from adjacent GM crops would affect the seed purity of a non-GM crop variety^4^. Therefore, it is necessary to research on the reliable detection of GM organisms, which is one of the most significant consumer concerns regarding food quality and safety.

Numerous techniques have been proposed for identification and classification of GM organisms, such as polymerase chain reaction (PCR)^5^,^6^, enzyme-linked immune sorbent assay (ELISA)^7^,^8^, microarrays^9^,^10^, electrophoresis^11^,^12^, biosensors^13^, chips^14^, mass spectrometry^15^ and chromatographic protein profiles^16^. However, most of these methods are destructive, time consuming, costly, difficult operations, and impossible for rapid on-site measurements. As non-destructive technologies, spectroscopic techniques are rapid and easy to operate without complicated sample preparations. Infrared spectroscopy^17^, near infrared (NIR)^18^,^19^,^20^,^21^, visible/near infrared (VIS-NIR)^22^, and multispectral imaging^23^,^24^ techniques combined with chemometric methods have shown their success in the rapid identification of GM organisms. Although many of the spectroscopic techniques mentioned above have been used to identify GM organisms, little attention has been paid to the use of terahertz (THz) spectroscopy for the detection of GM organisms.

THz spectroscopy usually refers to electromagnetic wave with the frequency of 0.1–10 THz (wavelength 30 μm-3 mm), and the band between microwave and infrared^25^. THz radiation is low-energy, non-ionizing and can penetrate a wide variety of non-conducting materials such as paper, wood, and plastic but it does not penetrate metal or water^26^. More importantly, theoretical studies show that most biological molecules, such as DNA components, protein and amino acids exhibit fingerprint spectra in the THz region^27^. Recently, THz spectroscopy together with chemometric methods is increasingly used in the fields of agricultural and food industry^27^,^28^,^29^. Moreover, it has also recently emerged as a powerful approach for the discrimination of different transgenic cottons and rice^30^,^31^,^32^. However, the defection of GM organisms using THz spectroscopy in previous studies needed to press samples into sheets or pellets, and up till now, there are only a few research reports about the application of THz spectroscopy technique for rapid and non-destructive detection of GM organisms without any sample preparation^33^.

Thus, the objective of the current study was to develop a non-disruptive method to discriminate among glyphosate-resistant, conventional and hybrid descendant seeds without sample preparation using THz spectroscopy combined with different chemometric methods including principal component analysis (PCA), least squares-support vector machines (LS-SVM) and PCA-back propagation neural network (PCA-BPNN).

## Results

### Spectra analysis

**Figure 1:**
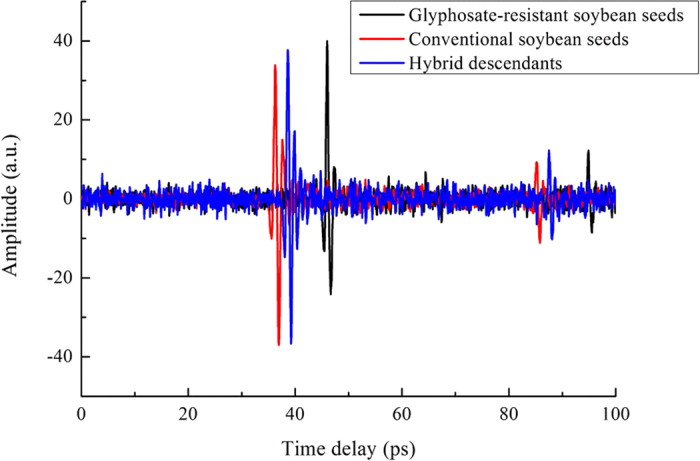
Average THz time-domain spectra of glyphosate-resistant (DP4546RR) and conventional (Wandou 28) soybean seeds and their hybrid descendants (DP4546RR × Wandou 28).

**Figure 2:**
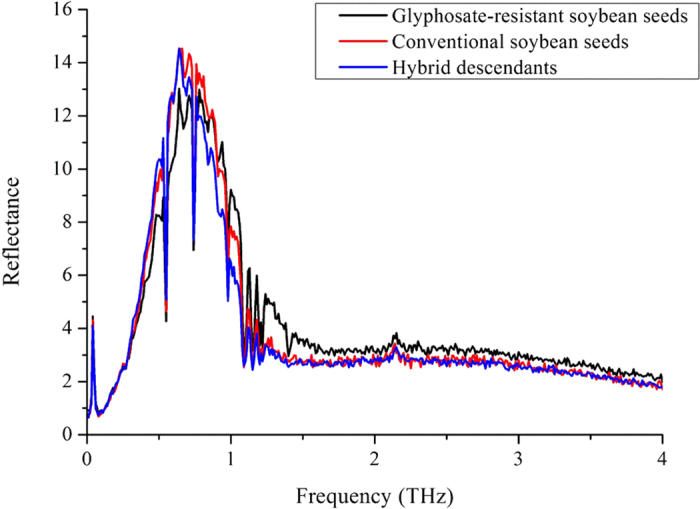
Average reflectance spectra of glyphosate-resistant (DP4546RR) and conventional (Wandou 28) soybean seeds and their hybrid descendants (DP4546RR × Wandou 28).

The average amplitudes of glyphosate-resistant (DP4546RR) and conventional (Wandou 28) soybean seeds and their hybrid descendants (DP4546RR × Wandou 28) in the time-domain spectra were shown in Fig. 1. Clearly, the general trend of all spectra was very similar, except for some subtle alterations. Although there was apparent time delay in time-domain spectra, no significant difference in the waveforms and the pulse amplitudes was observed among glyphosate-resistant and conventional soybean seeds and their hybrid descendants. Therefore, the frequency-domain spectra were obtained by transformation from time-domain spectra with fast Fourier transform (FFT) which was shown in Fig. 2. As shown in Fig. 2, these three classes of soybean seeds all had distinct absorption peaks at around 0.6 THz and 0.8 THz. The most obvious difference between 0.5 THz and 1.5 THz could be attributed to the chemical differences among glyphosate-resistant and conventional soybean seeds and their hybrid descendants. However, the spectra of these three classes of soybean seeds overlapped and were hard to differ from each other. Thus, it is difficult to discriminate these three classes of soybean seeds based on frequency-domain spectra with simple methods.

### Surface reflection intensity and penetration depth of soybean seeds

**Figure 3:**
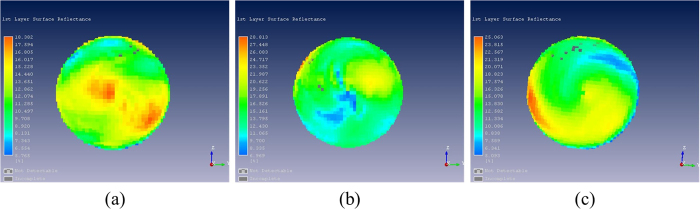
Surface reflection intensity of soybean seeds. **(a)** Glyphosate-resistant soybean seed; **(b)** Conventional soybean seed; **(c)** Hybrid descendants.

**Figure 4:**
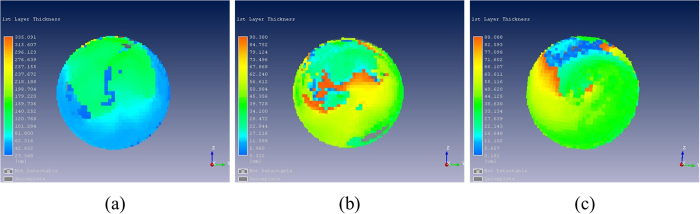
Penetration depth of soybean seeds. **(a)** Glyphosate-resistant soybean seed; **(b)** Conventional soybean seed; **(c)** Hybrid descendants.

Surface reflection intensity and penetration depth of glyphosate-resistant and conventional soybean seeds and their hybrid descendants were shown in Figs 3 and 4. Figure 3 showed that the surface reflection intensity of glyphosate-resistant soybean seed was higher than the non-transformed variety and the F1 progeny of the cross DP4546RR × Wandou 28. In addition, the penetration depth of glyphosate-resistant soybean seeds was relatively thinner than for seeds obtained from conventional variety Wandou 28 and progeny from crosses of DP4546RR × Wandou 28 (Fig. 4).

### Varieties discrimination

**Figure 5:**
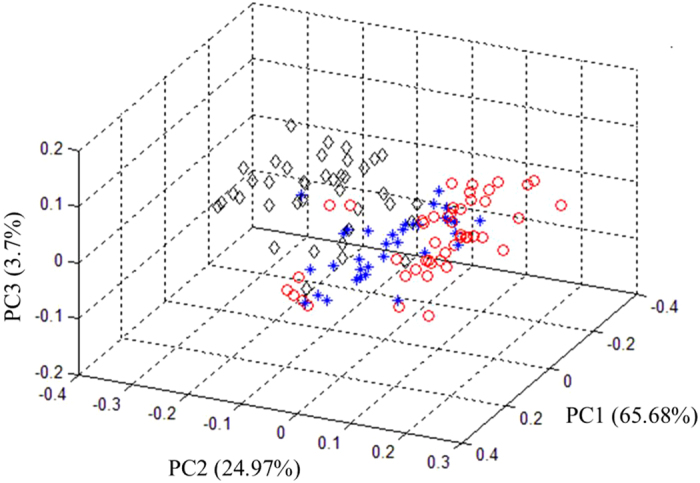
Three dimensional score plot of the first three principal components for the glyphosate-resistant (DP4546RR, ◊) and conventional (Wandou 28, *) soybean seeds and their hybrid descendants (DP4546RR × Wandou 28, ○).

PCA was performed initially to examine the qualitative difference of glyphosate-resistant and conventional soybean seeds and their hybrid descendants in principal component (PC) space. All the raw reflectance spectra obtained from the 60 glyphosate-resistant (DP4546RR), 60 conventional (Wandou 28) and 60 progeny seeds (DP4546RR × Wandou 28) were used for PCA. Figure 5 shows the three dimensional PC score plot of the samples. The results indicated that the initial three principal components (PCs), which account for 93% of the spectral variations (65.68%, 24.97%, and 3.7% for PC1, PC2, and PC3, respectively), allows for a clear differentiation among the three seed samples. Furthermore, these results suggested that discrimination among glyphosate-resistant, conventional and their hybrid descendant seeds was possible and that the different spectral attributes of these samples were associated with the characteristics of the seeds.

Back propagation neural network (BPNN) was performed on the first 20 PCs that contain more than 99.5% of the variation in the raw spectra data, which is defined as PCA-BPNN. In the PCA-BPNN method, to produce a high accuracy, the optimum values of the hidden nodes, the goal error and iteration times were determined to be 20, 1 × 10^−8^ and 800, respectively. With these optimal parameters, the soybean seeds could be classified and the discrimination results were listed in Table 1. Compared with the discrimination accuracies from the models using the raw spectra and the spectra with the first and second derivative pre-treatments, the one using the spectra with the standard normal variate (SNV) transformation pre-treatment achieved the best discrimination accuracies (the accuracies were 89.17% and 76.67% in calibration and validation set, respectively).

Least squares-support vector machine (LS-SVM) with the spectra range between 0.5 and 1.5 THz was used to build the calibration models. In the stage of model development using LS-SVM with radial basis function (RBF) kernel, the crucial step was the optimization of the meta-parameters *γ* and *σ*^2^ because their values determined the boundary complexity and the prediction performance. The optimal combination of (*γ*, *σ*^2^) for soybean seeds discrimination were found at the value of (0.25, 1) on the raw spectra, (6.9644, 0.015625) on the spectra with the first derivative, (2.82843, 0.0625) on the spectra with the second derivative, and (2, 0.0625) on the spectra with SNV, respectively. The discrimination results were also listed in Table 1. Similar to BPNN, the LS-SVM method using the spectra with SNV pre-treatment produced the best discrimination accuracies as compared with the raw spectra and the spectra with the first and second derivative pre-treatments. The discrimination accuracies in the calibration and validation set were 97.5% and 88.33%, respectively.

In order to analyse which seeds were more difficult to differentiate from the others, the best model using LS-SVM and the spectra with SNV pre-treatment were selected to assess its classification performance for differentiating the two parental varieties (DP4546RR and Wandou 28) and their offspring (DP4546RR × Wandou 28) of soybean seeds by using a confusion matrix. Table 2 lists the observation numbers, sensitivity and specificity of each set of seeds in validation data set. The highest number of misclassified seeds was observed between glyphosate-resistant soybean seeds and hybrid progeny, where three samples of glyphosate-resistant soybean seeds were wrongly classified as samples of hybrid descendants.

## Discussion

Different varieties of soybean seeds may have different chemical components and seed coat structure^34^,^35^,^36^. THz waves have the ability to penetrate a wide variety of materials, and vibration and rotational energy levels of most biological molecules (DNA, protein) are in the THz band. Thus, there was an apparent time delay in the time-domain spectra and different reflectance in the frequency-domain spectra of the glyphosate-resistant variety DP4546RR, the conventional variety Wandou 28 and their hybrid progeny seeds which were shown in Figs 1 and 2. In Fig. 1, there were significant differences in time delay among seeds from the two parental varieties and their progeny, which might be attributed to differences in chemical composition and coat structure of the seeds. Furthermore, as shown in Fig. 2, these three sets of soybean seeds showed a similar trend in absorption spectra between 0.1 THz and 4 THz, especially from 0.5 THz to 1.5 THz. Apparent absorption peaks at around 0.6 THz and 0.8 THz were observed which may be due to the water absorption of soybean seed samples in the THz region^37^. In Figs 3 and 4, when comparing the surface reflection intensity and the penetration depth of these three types of seeds, the hardness or thickness of the seed coat of the glyphosate-resistant soybean variety seem to be lower/finer than the Wandou 28 variety with offspring of the two varieties showing intermediate hardness/thickness as expected. From the Table 2, the highest frequency of misclassification of seeds was observed between glyphosate-resistant soybean seeds and hybrid descendants, which might be due to the similar chemical components of these two varieties.

Chemometrics can improve the understanding of chemical information and correlate quality parameters or physical properties using mathematical and statistical methods. The advanced methods can enable a more thorough scientific characterization of samples than the information obtained from simple analysis. In this study, the classification of the transgenic and conventional soybean seeds and their hybrid descendants with THz spectroscopy can be seen as a high-dimensions and non-linear pattern recognition problem. Each chemometric method has its own advantages and applications. As seen from Table 1, the best discrimination accuracies were obtained using LS-SVM method combined with the spectra with SNV pre-treatment for discrimination of glyphosate-resistant and conventional soybean seeds and their hybrid descendant. The reasons may be as follow: (1) SVM is specifically designed to operate in high-dimensional characteristic space with fewer training variables or samples; (2) It has effective performance for multivariate function estimation especially for non-linear classification.

## Methods

### Soybean seed samples

Glyphosate-resistant GM soybean seeds (ROUNDUP READY soybean DP4546RR, Monsanto Canada Inc.), conventional soybean seeds (Wandou 28), and their hybrid descendants (DP4546RR × Wandou 28) were provided. The F1 progeny of the cross DP4546RR × Wandou 28 was resistant to glyphosate, but was not genetically homogenous. In the current study, three varieties (glyphosate-resistant and conventional soybean seeds and their hybrid descendants) were defined. A total of 180 soybean seed samples were randomly divided into a calibration set (2/3, including 40 seeds of each sample) and a validation set (1/3, including 20 seed samples of each plant variety).

### THz spectroscopy imaging system

THz time-domain spectra of all the intact samples, including 60 glyphosate-resistant (DP4546RR) and 60 conventional (Wandou 28) soybean seeds and 60 their hybrid descendants (DP4546RR × Wandou 28), were collected by a Fiber-Coupled (FiCO) terahertz spectroscopy imaging system (Zomega Terahertz Corporation, East Greenbush, NY, USA) with independent fiber-coupled emitter and receiver heads. The system is designed to perform both transmission and reflection spectroscopy and imaging measurements in the range from 0.1 THz to 4 THz, with a waveform acquisition speed up to 500 Hz. For this study, the measurements were conducted in a reflection mode. All of the spectra were collected after the spectroscopy system warmed up for half an hour to reach a stable state. The measurements were carried out under dry air. Firstly, a measure of the noise level was obtained by blocking the path of the THz beam with a metallic plate. The measured signal provides the spectral characteristics of the noise of the system. The noise level allows determining the dynamic range of the system as a function of frequency as the difference (in dB) between the maximum signal available and the noise level. For FiCO, the peak dynamic range (in power) is around 50 dB. And then, each soybean seed sample was measured.

### Spectra data pre-treatment

For each sample, the acquired time-domain spectra data were transformed into the frequency-domain spectra using the FFT. The shape of the time-domain data is a pulse with duration of the order of the picosecond, which is equivalent to terahertz in the frequency-domain. In this study, derivative processes, including the first and second derivative, and SNV were selected as spectra pre-treatment methods.

### Chemometric methods

Multivariate analysis including PCA, LS-SVM, and PCA-BPNN were selected as the chemometric methods in present study to classify and screen these three varieties. All the selected chemometric methods were compared and the accuracy results obtained from the calibration and validation set were summarized accordingly. All of these chemometrics analysis and statistics were performed using the commercial software Matlab 2009 (The Mathworks Inc., Natick, MA, USA), Image J 1.48 and Origin 8.5.

## Additional Information

**How to cite this article**: Liu, W. *et al*. Discrimination of transgenic soybean seeds by terahertz spectroscopy. *Sci. Rep.* **6**, 35799; doi: 10.1038/srep35799 (2016).

**Publisher’s note:** Springer Nature remains neutral with regard to jurisdictional claims in published maps and institutional affiliations.

## Figures and Tables

**Table 1:** Comparison of discrimination performance obtained with PCA-BPNN and LS-SVM methods and the THz spectra with different pre-treatments.

Chemometric methods	Data pre-treatment	Number of misclassified samples in calibration set	Number of misclassified samples in validation set	Accuracy in calibration set (%)	Accuracy in validation set (%)
LS-SVM	no	4	10	96.67	83.33
	1^st^ derivative	2	12	98.33	80
	2^st^ derivative	2	13	98.33	78.33
	SNV	3	7	97.5	88.33
PCA-BPNN	no	12	15	90	75
	1^st^ derivative	16	17	86.67	71.67
	2^st^ derivative	19	20	84.17	66.67
	SNV	13	14	89.17	76.67

**Table 2:** Matrix detailing the classification results of seeds from glyphosate-resistant (DP4546RR), conventional (Wandou 28) and the progeny (DP4546RR × Wandou 28) using the LS-SVM method and the THz spectra with SNV pre-treatment.

Actual class	Predicted class		
	Glyphosate-resistant	Conventional	Hybrid descendants
Glyphosate-resistant	16	1	3
Conventional	0	18	2
Hybrid descendants	1	0	19
Sensitivity (%)	80	90	95
Specificity (%)	100	95	87.5
